# Assessing the Impact of Higher Levels of CO_2_ and Temperature and Their Interactions on Tomato (*Solanum* *lycopersicum* L.)

**DOI:** 10.3390/plants10020256

**Published:** 2021-01-28

**Authors:** Tejaswini C. Rangaswamy, Shankarappa Sridhara, Nandini Ramesh, Pradeep Gopakkali, Diaa O. El-Ansary, Eman A. Mahmoud, Shaimaa A. M. Abdelmohsen, Ashraf M. M. Abdelbacki, Hosam O. Elansary, Amal M. E. Abdel-Hamid

**Affiliations:** 1 Center for Climate Resilient Agriculture, University of Agricultural and Horticultural Sciences, Shivamogga 577204, Karnataka, India; cr.tejaswini9@gmail.com (T.C.R.); nandinianuram@gmail.com (N.R.); g.pradeep76@gmail.com (P.G.); 2 Precision Agriculture Laboratory, Department of Pomology, Faculty of Agriculture (El-Shatby), Alexandria University, Alexandria 21545, Egypt; diaaagri@hotmail.com; 3 Department of Food Industries, College of Agriculture, Damietta University, Damietta 34511, Egypt; emanmail2005@yahoo.com; 4 Physics Department, Faculty of Science, Princess Nourah bint Abdulrahman University, Riyadh 84428, Saudi Arabia; shamohamed@pnu.edu.sa; 5 Plant Pathology Department, Faculty of Agriculture, Cairo University, Cairo 12613, Egypt; amaeg@hotmail.com; 6 Plant Production Department, College of Food and Agriculture Sciences, King Saud University, Riyadh 11451, Saudi Arabia; 7 Floriculture, Ornamental Horticulture and Garden Design Department, Faculty of Agriculture (El-Shatby), Alexandria University, Alexandria 21545, Egypt; 8 Department of Biological and Geological Sciences, Faculty of Education, Ain Shams University, Cairo 21974, Egypt; amelmohamed@edu.asu.edu.eg

**Keywords:** elevated CO_2_, elevated temperature, tomato, open top chamber

## Abstract

Climate change has increasing effects on horticultural crops. To investigate the impact of CO2 and temperature at elevated levels on tomato production and quality of fruits an experiment was conducted by growing plants in open top chambers. The tomato plants were raised at EC_550_ (elevated CO_2_ at 550 ppm) and EC_700_ (elevated CO_2_ at 700 ppm) alone and in combination with elevated temperature (ET) + 2 °C in the open top chambers. These elevate CO_2_ and temperature treatment effects were compared with plants grown under ambient conditions. Outcome of the experiment indicated that growth parameters namely plant stature in terms of height (152.20 cm), leaf number (158.67), canopy spread (6127.70 cm^2^), leaf area (9110.68 cm^2^) and total dry matter (223.0 g/plant) were found to be high at EC_700_ compared to plants grown at ambient conditions in open field. The plants grown at EC_700_ also exhibited significantly higher number of flowers (273.80) and fruits (261.13), more fruit weight (90.46 g) and yield (5.09 kg plant^−1^) compared to plants grown at ambient conditions in open field. The percent increase in fruit yield due to EC varied from 18.37 (EC_550_) to 21.41 (EC_700_) percent respectively compared to open field and the ET by 2 °C has reduced the fruit yield by 20.01 percent. Quality traits like Total Soluble Solids (3.67 °Brix), reducing sugars (2.48%), total sugars (4.41%) and ascorbic acid (18.18 mg/100 g) were found maximum in EC_700_ treated tomato than other elevated conditions. Keeping quality was also improved in tomato cultivated under EC_700_ (25.60 days) than the open field (17.80 days). These findings reveal that CO_2_ at 700 ppm would be a better option to improve both quantitative as well as qualitative traits in tomato. Among the combinations, EC_550_ + 2 °C proved better than EC_700_ + 2 °C with respect to yield as well as for the quality traits. The tomato grown under ET (+2 °C) alone recorded lowest growth and yield attributes compared to open field conditions and rest of the treatments. The positive influence of EC_700_ is negated to an extent of 14.35 % when the EC_700_ combined with elevated temperature of + 2 °C. The present study clearly demonstrates that the climate change in terms of increased temperature and CO_2_ will have a positive effect on tomato by way of increase in production and quality of fruits. Meanwhile the increase in EC beyond 700 ppm along with ET may reduce the positive effects on yield and quality of tomato.

## 1. Introduction

Carbon dioxide levels are increasing drastically in the atmosphere contributing a major share to global warming. In many countries, agriculture is the principal source of revenue and climate is a crucial factor for crop development. Global temperature is found to increase due to various human activities and interventions increasing the emission of methane (CH_4_), Nitrous oxide (N_2_O) and carbon dioxide (CO_2_) which are considered as greenhouse gases. According to Inter-governmental Panel on Climate Change [1] projections with respect to RCP 6.0 (Representative Concentration Pathway) indicate continuous global warming where CO_2_ levels may rise to 670 ppm by 2100 and also the global temperature is predicted to increase by about 3–4 °C. The National Oceanic and Atmospheric Administration [2] reported the present level of atmospheric CO_2_ concentration to be 414 ppm and are expected to rise over 700 ppm by the end of this century if no proper measures are adopted. The two important climatic factors like temperature and carbon dioxide are inter-related with one another affecting the whole biosphere [3]. Plants do respond positively as well as negatively to the elevated levels of CO_2_ and temperature based on their photosynthetic pathway. Elevated levels of CO_2_ known to increase the rate of photosynthesis as well as biomass accumulation. Meanwhile increase in temperature especially in tropical countries known to increase in transpiration rate and stomatal conductance apart from increasing photorespiration and maintenance respiration in C_3_ plants. Hence it is essential to study their impact individually and in combination as to know how they affect vegetable crops and their production.

Effect of elevated carbon dioxide (EC) on nutritional quality of vegetables *viz*., lettuce, tomato and potato were assessed by Dong et al. [4] and the results revealed that glucose, fructose, total flavonoids, phenols, soluble sugar, antioxidants, ascorbic acid as well as calcium concentration in the vegetable’s edible part increased due to EC level. On contradictory, EC at 500 and 700 ppm lowered the content of flavonoids, total soluble solids, titrable acidity and phenols in tomato [5]. Similar attempts were made to know the impact of elevated temperature (ET) on tomato fruit yield and quality traits wherein, high temperatures reduced the lycopene content, fruit quality and mineral content of tomato [6] and have a negative relationship with tomato yield [7]. Fruit number, fruit set and development were reduced when expose to higher temperatures [8,9]. But the studies conducted to understand the response of vegetables especially tomato to EC, ET and their combined effects in terms of growth, yield and quality are lacking.

Interactive studies on effect of EC and ET on varied crop species reveal that, number of tillers and filled grains increased in case of cereals under EC however same parameters decreased at elevated temperatures [1]. EC coupled with ET (+2–4 °C) enhanced plants uptake of CO_2_ which in turn resulted in increased photosynthesis as the accumulation of carbohydrates were reduced due to high temperature levels [10]. Combination of CO_2_ (720 ppm) and temperature (+5 °C) at elevated levels decreased number of strawberry fruits and yield while, the strawberries grown under EC_720_ alone showed significant increase in fruit yield [11]. Lenka et al., 2017 [12] suggested that in case of soybean, seed index as well as grain yield had increased significantly under EC and ET combination. This urges to understand the consequences of CO_2_ and temperature levels at elevated conditions alone and their interactive reactions on growth attributes as well as on yield and quality of crops by field experimentation. Among the various vegetable crops, tomato is being cultivated in all the regions of tropical zones. It is a store house of antioxidants, vitamins, organic acids, minerals with 2.5 percent of total sugar content, 16.0–65.0 mg 100 g^−1^ of ascorbic acid, carbohydrates along with fat content of 4 and 0.3 percent respectively [13]. Various research reports reveal that elevated concentrations of CO_2_ may enhance yield levels of C_3_ crops wherein the other biotic or abiotic stresses are being absent [14]. Generally, vegetables are highly susceptible to climate change. Among various stressful conditions, high atmospheric CO_2_ concentration and high temperatures tend to be most important. Being a C_3_ crop, the growth and yield parameters of tomato have shown a positive response to the increase in CO_2_ concentration.

Carbon dioxide is the primary raw material of photosynthesis, an increase in its concentration increases the photosynthetic rate and thus it also increases the productivity and yield. In general CO_2_ concentration of 700 ppm to 1000 ppm enhances the vegetable yield [15]. We hypothesize that tomato being a C_3_ crop may benefit from increase in the CO_2_ present in the atmosphere along with interactive effect of EC and ET may affect the yield and quality. The present study was carried out to explore the impact of EC, ET alone and their combination on tomato plants and the extent to which enhanced CO_2_ concentration negotiates the harmful effects of elevated temperature resulting in higher growth and yield especially in tropical countries like India. Apart from this we also aimed at assessing these effects on quality parameters of tomato fruits.

## 2. Materials and Methods

An experiment was conducted at field during *kharif* 2019 at Center for Climate Resilient Agriculture, University of Agricultural and Horticultural Sciences, Shivamogga, Karnataka, India which is situated at 13°58′ N 75°34′ E at an elevation of 615 m. The soil of the experimental area was found to be sandy loam textured with neutral pH of 6.60 having low nitrogen content (248 kg/ha), high Phosphorus (70.57 kg/ha) and medium Potassium (315.12 kg/ha). About 940.5 mm of precipitation was recorded at the time of experiment which was more than the normal rainfall (435.8 mm). Mean minimum and maximum temperature were 17.6 °C and 30.7 °C, respectively during the crop growth period. Experiment was carried out in Randomized Complete Block Design (RCBD) with seven treatment combinations involving three varied levels of CO_2_ concentrations *viz.*, ambient (414 ppm), 550 and 700 ppm and two temperature levels *viz.*, ambient and elevated (+2 °C). The experiment consists of three replications. There were six open top chambers (OTC) used for experimentation. One OTC was maintained under ambient conditions of CO_2_ and temperature (reference OTC), two with elevated conditions of CO_2_ alone (EC_550_ and EC_700_), two with combination of elevated CO_2_ and elevated temperature (EC_550_ + 2 °C and EC_700_ + 2 °C), one with elevated temperature (ET) + 2 °C alone and one open field plot.

**Figure 1:**
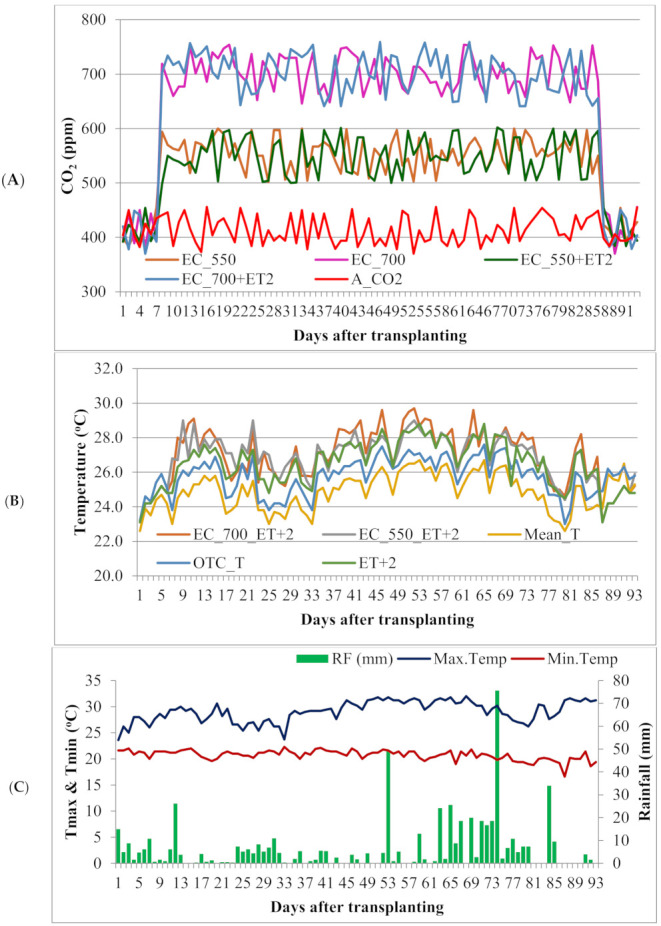
Daily CO_2_ (**A**), Temperature (**B**) values recorded under each of the OTCs during the experimental period and the actual weather parameters prevailed during the experimental period (**C**). EC_550 (Elevated CO_2_ at 550 ppm), EC_700(Elevated CO_2_ at 700 ppm), EC_550+ET2 ((Elevated CO_2_ at 550 ppm + Elevated temperature by 2 °C), EC_700+ET2 ((Elevated CO_2_ at 700 ppm + Elevated temperature by 2 ^o^C), A_CO_2_ (Ambient CO_2_), OTC_T (Temperature within the OTC), Mean_T (Average atmospheric temperature), ET + 2 (Elevate temperature by 2 °C).

To maintain the above described conditions, pure CO_2_ gas was supplied to the chambers and maintained at set levels using Non-dispersive infrared (NDIR) sensors-based CO_2_ gas analyzer. The opening and closing of the valves inside the chamber were regulated on the basis of the set level of the CO_2_ for a particular OTC which was regulated by computer through linked Supervisory Control and Data Acquisition (SCADA) system. The CO_2_ concentration inside the OTC was maintained to the desired level by injecting CO_2_ gas routinely using cylinders containing CO_2_ at 7.30 a.m. in the morning to 5.30 p.m. starting from seven days after transplanting till seven days prior to harvest of the crop. The desired level of temperature in the chamber was maintained through the infrared heaters mounted all around the perimeter of OTC. The temperature was set at + 2 °C than the ambient temperature. Inside and outside temperature could be sensed by the digital temperature controller and as the temperature increased more than + 2 °C, heaters would switch off automatically. The data pertaining to daily measured temperature and CO_2_ within the OTCs (mean over the time of a day during the elevations were imposed, i.e., 10 h) as well as the weather parameters prevailed during the experimental period (mean over 24 h) are presented in Figure 1.

The land inside the OTC was thoroughly digged manually up to 30 cm depth, and the resultant soil was brought to fine tilth after removing the weeds and stubbles a fortnight prior to planting. Farm yard manures at the rate of 25 tonnes ha^−1^ was applied 15 days before transplanting and incorporated in to the soil. Transplanting of 30 days old tomato seedlings (Arka Rakshak) was done in the open top chambers (dimension of 5 × 5 × 3 m). Each OTC had 25 plants with a spacing of 90 cm × 90 cm. Recommended dose of fertilizers 180 kg Nitrogen (Urea), 120 kg Phosphorous (Single Super Phosphate), and 150 kg Potassium (Muriate of Potash) per hectare were applied in three split doses Basal dose (50% N, 25% P and K) was applied at 4 days after transplanting whereas, second dose (25% N and 50% P and K) and third dose (25% N, P and K) was applied at 30 and 50 DAT respectively. Micronutrients were supplemented by spraying vegetable special (Zinc: 225 ppm, Boron: 50 ppm, Manganese: 42.5 ppm, Iron: 105 ppm and Copper: 5 ppm) at 4 g L^−1^ during 25 DAT, flowering and fruit initiation stages. All the agronomic practices were carried out uniformly to raise the seedlings in the open top chambers.

### 2.1. Growth Characteristics

Growth attributes *viz.*, number of leaves/plants, plant height, and canopy spread were recorded on randomly labelled five plants in each treatment. Canopy spread was measured in both North-South as well as East-West directions at 30 days interval and expressed in cm^2^. Measurement of total area of leaf (cm^2^) was estimated with standard LI-COR leaf area meter (Model LI-3100, LICOR Inc. Lincoln, NE, USA). Total plant dry matter was recorded separately from each OTC at harvest. The dry weight of roots, stem and leaves was recorded by drying the plant parts keeping in hot air oven at a temperature of 65 °C till constant weight is obtained and finally total dry weight per plant was recorded.

### 2.2. Yield Characteristics

The numbers of flowers were counted starting from first flower blooming till the fruit initiation in all the plots from five randomly tagged plants. Number of flowers were added successively and expressed as cumulative flower numbers plant^−1^. Similarly, total number of fruits were recorded from every treatment and expressed as cumulative fruits plant^−1^. The fully matured fruits were harvested whenever they reach the ripening stage. The total fruit weight obtained from first to final harvest was recorded which gives average yield plant^−1^ in kilograms. Also, fruit weight of five selected plants was recorded from all the treatments and then average fruit weight (g) was calculated.

### 2.3. Quality Traits

#### 2.3.1. Keeping Quality (Days)

The harvested fruits were kept under normal room temperature and the numbers of days from harvest till the fruits are unsuitable for consumption (whenever the fruit skin becomes softer and develops wrinkles) was recorded and the mean number of days was calculated as described by [13].

#### 2.3.2. Total Soluble Solids (°Brix), Titratable Acidity (%) and pH

The total soluble solids content of fruit was estimated using portable hand refractometer. Titratable acidity of tomato was estimated as per the protocol of AOAC, 2000 [16]. As per the procedure, 5 g of tomato juice extract was diluted in 25 mL of distilled water and titrated against 0.1 N sodium hydroxide (NaOH) to pH 8.1 using 1% phenolphthalein indicator.

The pH value of tomato juice was estimated using a digital pH meter [16].

#### 2.3.3. Fruit Firmness (kg/cm^2^)

The firmness of tomato fruits was assessed using an instrument i.e., Penetrometer (Fruit pressure tester FT-327, QA Supplies LLC, Norfolk, VA, USA). The procedure was by rupturing the fruit with plunger in four places opposite to each other along the radial axis. The pressure exerted by penetrometer for rupturing the fruit determines the fruit firmness [13].

#### 2.3.4. Ascorbic Acid (mg/100 g), Lycopene Content (mg/100 g) and Carotenoid Content (μg/g)

Ascorbic acid present in fruits was measured using the volumetric method. Tomato sample of 5 mL (prepared by mixing 2.5 g of fruit in 50 mL 4 % oxalic acid) was titrated against the 2,6-dichlorophenol indophenol dye to the pink colour end point. The resultant ascorbic acid content was expressed in mg/100 g [17].

The lycopene and carotenoid content of tomato were estimated by macerating one gram of tomato pulp in pestle and mortar using acetone until the colourless extractant was obtained. Then the contents were transferred to a separating funnel which contained acetone extract, 100 mL distilled water and 15 mL of hexane. This mixture was rotated well and allowed to settle some minutes until two differentiating layers were formed. The upper hexane layer containing pigments were collected in a 25 mL volumetric flask and the volume was made up. Then the aliquot of one ml was further diluted to 5 mL with hexane and absorbance was recorded at 503 nm for lycopene and at 470 nm for carotenoid using spectrophotometer (Perkin Elmer, UV-Vis, LAMBDA 365, Waltham, MA, USA) [18].

#### 2.3.5. Sugar Content (%)

Sugars (Total sugar, reducing and non-reducing sugar) present in tomato fruit were estimated using the procedure given by the Lane and Eynon method [19].

Reducing and total sugars were determined by taking tomato sample of 5 g into a beaker containing 100 mL distilled water. The solution was continuously stirred and filtered through Whatman No. 1 filter paper into a 250 mL volumetric flask. 100 mL of the solution was pipetted into a conical flask to which 10 mL of diluted HCl was added and boiled for about 5 min. After cooling, the solution was neutralized with 10% NaOH to phenolphthalein end point and the volume was made up to 250 mL. Then it was titrated against Fehling’s solution and the sugar content was expressed in percent. Non-reducing sugar was measured by subtracting the reducing sugar out of total sugar content.

### 2.4. Analysis of Data Through Statistical Procedure

The data obtained during the experiment was analysed statistically by using SPSS software V25.0 with assistance of colleagues from Princess Nourah bint Abdulrahman University. One-way analysis followed by post hoc Tukey’s Honest Significant Difference (HSD) test is employed for mean comparison apart from using Least Significant Difference (LSD). The data are presented as mean ± Standard Error (SE). The level of significance used was at *p* = 0.05. Correlation analysis was carried out to know the relationship between yield and quality traits of tomato. Pearson correlation coefficients were computed by using Performance Analytics package V2.0.4 in R environment. The significance of the correlation coefficients was tested by employing t-test.

## 3. Results and Discussion

### 3.1. Growth Attributes of Tomato

An assessment of data on impact of CO_2_ and temperature at elevated levels on tomato at 90 days after transplanting (DAT) indicates that significant improvement in all the growth parameters were observed under elevated EC_700_ followed by EC_550_ and combination of EC_550_ and ET + 2 °C (Table 1). Meanwhile these growth parameters reduced due to exposure of plants to higher temperature by 2 °C. The plant height of tomato improved with both elevated concentrations of CO_2_ compared to ambient conditions in reference OTC and open field. Significantly higher plant height was recorded (152.20 cm) in EC_700_ as compared to open field (138.13 cm). This variation may be due to an increased rate of net photosynthesis under elevated CO_2_ condition. Jones [20] documented that elevated CO_2_ improves plant growth and biomass through enhanced photosynthetic rate in leaves, water use efficiency and decreased transpiration. Increased CO_2_ level has also been found to increase leaf size [21]. Further there exists a differential response by plants to increased levels of CO_2_ depending upon the photosynthetic pathway. Tomato being a C_3_ plant benefited by its exposure to elevated levels of CO_2_ as increase in CO_2_ concentrations stimulates the rate of photosynthesis by increasing the concentration gradient from air to leaf apart from decreasing the photorespiration and reduced expression of Rubisco. Similar reports are quoted by [12] who showed the positive effect of higher CO_2_ coupled with higher temperature on plant height of soybean. In tomato, every leaf assimilates to develop into fruit. Thus, production of leaves serves as an important phenomenon during fruit development. The number of leaves and canopy spread was significantly increased in EC_700_ (158.67 and 6127.70 cm^2^ respectively). However, subsequent higher values were noticed in EC_550_ (147.33 and 5748.00 cm^2^ respectively). The increased number of leaves in the current investigation is in corroboration with Conroy et al. [22] who reported that *Pinus radiata* plants under higher CO_2_ concentration have higher number of leaves in the broader canopy. This type of related result was noticed in tomato by [5,23,24].

An increase in the total leaf area at higher CO_2_ levels has been observed in the present study. Highest leaf area per plant was observed at EC_700_, where it was 42.39 and 5.19% more than control and elevated EC_550_, respectively (Table 1). The enlarged leaf area may be attributed to an increase in photosynthesis, cell division and differentiation, resulting in an increased rate of leaf area expansion [25]. These kinds of results were also noticed by [26] who observed increase in leaf area of carrot at elevated CO_2_ compared to ambient condition due to stimulation of leaf area expansion than increase in leaf number. This was consistent with the studies of [27] in maize and [12] in soybean.

**Figure 2:**
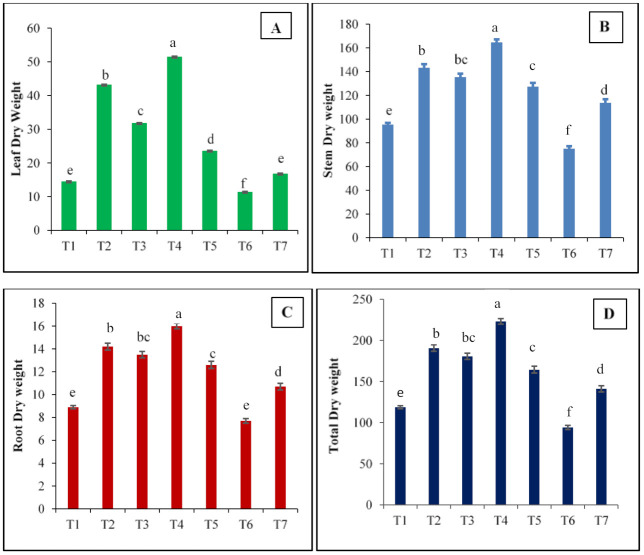
Effect of different treatments of CO_2_ and temperature on leaf dry weight (**A**), stem dry weight (**B**), root dry weight (**C**), and total dry weight (**D**) of tomato at harvest. T1—ambient CO_2_ and temperature, T2—550 ppm, T3—550 ppm + 2 °C, T4—700 ppm, T5—700 ppm + 2 °C, T6—Elevated temperature (+2 °C), T7—open field (control). Vertical bars indicate the Standard Error. Vertical bars with same letter(s) do not differ significantly at *p* = 0.05 level as per Tukeys HSD.

Tomato plants grown at EC_700_ recorded higher leaf, stem, root, and total dry matter than the plants grown at EC_550_ and ambient CO_2_ concentration. Higher total dry matter (223.0 g plant^−1^) at harvest is due to its higher leaf dry weight (46.4 g plant^−1^), stem dry weight (160.6 g plant^−1^) and root dry weight (16.0 g plant^− 1^). Production of dry matter increased with the age of plants and the maximum was recorded at harvest (Figure 2). Exposure of plants to higher temperature by 2 °C has resulted in the decrease of biomass accumulated in different parts of tomato. The reduction in biomass due to elevated temperature may be attributed to increase in transpiration rate as well as stomatal conductance, maintenance respiration and phot respiration [28,29]. Enhanced CO_2_ concentration promotes plant growth with a greater number of branches and leaves which enhances accumulation of dry matter in different plant parts compared to open field conditions. The increase in the biomass produced in different parts under elevated CO_2_ is attributed to increase in the activity of RuBP (carboxylase)/Rubisco (oxygenase) activity resulting in enhanced photosynthesis [30]. The reports of [31] also showed that tomato seedlings grown at EC_675_ recorded increased biomass of 37%of leaf, 39% of root, 53% stem and 41% of total biomass. Aien et al. (2013) [32] reported the increase in biomass of potato grown under elevated CO_2_ by 35.8 % than the ambient CO_2_ conditions. Enrichment of CO_2_ resulted in increased root dry weight in tomato and pepper by 49 and 62 %, respectively [33]. Desjardins et al. (1990) [34] reported that elevated CO_2_ had a positive effect on shoot and root dry weights of asparagus. The results reported by [35] in lettuce, [36] in mungbean, [37] in cucumber and [38] in sweet pepper also shows that elevated CO_2_ enhanced the production of dry matter positively.

### 3.2. Yield and Yield Attributes

With the increase in CO_2_ concentration, the number of flowers and fruits per plant increased significantly, which in turn lead to higher fruit yield per plant at different CO_2_ concentrations *viz.,* EC_700_ and EC_550_ (Table 2). Significantly higher flower number (273.80) and fruit number (261.13) was recorded under EC_700_ as compared to other elevated conditions alone or in combination. The growth promoting effects of increased CO_2_ concentration might have attributed to higher number of flowers and fruits plant^−1^. The increase in flower number as well as fruits with increased CO_2_ concentration was due to increased sink strength more than source strength in tomatoes. More carbohydrates may be accumulated in fruits during the fruit development stage, resulting in higher yields [39]. Similarly, 21.5% and 41% increase in total yields of tomato were reported by [40] and [41], respectively. Thus, CO_2_ at higher concentrations under changing climatic conditions could attribute to higher yields.

The significant increase in growth attributing characters *viz.,* leaf area, plant length (height) and dry weights in turn resulted maximum total yield (Table 1 and Table 2 and Figure 1). The tomato plants in EC_700_ recorded highest fruit yield per plant (5.09 kg) as well as maximum fruit yield per hectare (54.11 t/ha) as compared to open field condition (3.67 kg/plant and 44.57 t/ha respectively) which may be evident due to improved growth and reproductive characters at higher levels of carbon dioxide. Among the combinations, EC_550_ + 2 °C recorded more fruit yield (4.33 kg/plant and 52.09 t/ha) than EC_700_ + 2 °C (4.21 kg/plant and 46.34 t/ha). Lowest fruit yield was recorded by plants raised under elevated temperature (+2 °C) alone. The percent increase in fruit yield due to EC_550_ and EC_700_ is 18.37 and 21.41 percent respectively compared to open field conditions. Men while the positive effect of EC either at 550 or 700 ppm has reduced the fruit yield by 1.49 and 17.43 percent respectively whenever the temperature in increased by 2 °C. This clearly indicates the positive effects of EC are reduced by ET. An increase of 0.19% per 1 ppm rise in CO_2_ for the yield of radish as reviewed by [42] and an increase of 0.15% per 1 ppm rise in CO_2_ for the yield of carrot [26] was observed at elevated CO_2_. The yield of tomato (5.09 kg/plant) increased in terms of fruit number plant^−1^ (261.13) and fruit weight (90.46 g) with EC_700_. Craigon et al. (2002) [43] found that tuber number of potatoes was increased under high CO_2_ concentration. Increased fruit size is the resultant of more carbohydrates accumulation in fruits due to enhanced photosynthetic rate in a high CO_2_ environment [40]. In the present study, fruit weight was increased at enhanced levels of atmospheric CO_2._ A similar research carried out by scientists suggests that, tomato cultivated under higher concentrations of atmospheric CO_2_ conditions produced tomato fruits with more weight than the tomato grown under normal CO_2_ levels [44,45,46]. The excessive accumulation of carbohydrates due to elevated CO_2_ might have been contributed to productivity through development of new sinks which results in increased yield [47,48]. An increase in CO_2_ concentration increased the potato yield by 30.8% over control [32]. This shows that, the tomato produced under changing climate scenarios will be benefitted due to higher levels of CO_2_ by masking the adverse effects of higher temperature levels and thus increase in production may be expected.

### 3.3. Quality Traits of Tomato

Quality assessment of tomato fruits reveal that keeping quality (25.60 days), TSS (3.67 °Brix), reducing sugars (2.48%) as well as total sugar content (4.41%) were found enhanced in tomato grown under EC_700_ followed by EC_550_ compared to ambient conditions of CO_2_ and temperature at OTC and open field. Whereas, the reducing sugars were lower in ET (+2 °C) with 1.64 % (Table 3). In tomato, reducing sugars contributes 95 per cent of the total sugars, whereas, non-reducing sugars constitute a minimal amount [45]. Similar findings were reported by [49,50,51] who opined that quality was improved in terms of total sugars, reducing and non-reducing sugars in tomato grown under higher CO_2_ levels than the plants grown at ambient CO_2_ concentrations. An increase in TSS with elevated CO_2_ is in agreement with [52], who reported that exposure of plants to 1000 ppm CO_2_ increases the TSS concentration when compared to ambient CO_2_ (340 ppm) conditions. Sucrose is a main product of photosynthesis and improved photosynthetic rate under enhanced CO_2_ conditions resulted in increased production of carbohydrates and their accumulation [53]. The reduction in the sugar content under elevated temperature conditions might be attributed to utilization of sugars towards maintenance respirations as well towards the energy used for transpiration.

Ascorbic acid content (18.88 mg/100 g) and titratable acidity (0.27%) was found more in EC_700_ with lower pH of 4.08. Islam et al. (1996) [45] showed more ascorbic acid content at elevated CO_2_ condition and at different maturity degrees. Such studies are in agreement with the research works conducted by [54] and [55] in sour orange and strawberry respectively grown at 700 ppm of CO_2_ in OTCs.

Carotenoid and lycopene contents were 14.53 µg g^−1^ and 9.36 mg 100 g^−1^ higher in EC_550_ respectively however, found lower in ET (+2 °C) with 9.59 µg g^−1^ and 5.15 mg 100 g^−1^ respectively (Table 4). Such findings agree with [56] in tomato, who observed significantly lower lycopene content at 700 ppm of CO_2_. The effect of higher CO_2_ concentration on lycopene content is variable due to the effects of temperature on lycopene synthesis [57].

### 3.4. Relationship Between Yield and Quality Traits of Tomato

The relationship between yield and quality traits of tomato grown under elevated CO_2_ and temperature levels alone and their combinations was analysed by carrying out the correlation studies which revealed that yield of tomato was statistically significant and positively correlated with TSS (0.89 ***), titratable acidity (0.84 ***) and ascorbic acid (0.94 ***). The other quality parameters *viz*., reducing sugar, non-reducing sugar showed some positive association with the fruit yield of tomato whereas, pH showed negative correlation with fruit yield of tomato (Table 5). Thus, TSS, titratable acidity and ascorbic acid can be considered for improving the yield of tomato genotypes with better quality. Similar kind of association between yield and quality traits of tomato was reported by [58,59,60,61].

## 4. Conclusions

The present investigation shows that plant height and leaf number at elevated CO_2_ significantly increased irrespective of the concentration. Concomitantly the leaf area and canopy spread were also increased with EC_700_ than open field conditions. Plants adapted to higher concentrations of CO_2,_ high temperature alone and their combinations resulted in the higher biomass of all the plant parts. Overall, the elevated CO_2_ condition counteracted the high temperature effect thereby resulting in increased number of flowers as well as fruits per plant with increased yield. The traits such as keeping quality, TSS, reducing sugars, total sugars, ascorbic acid content and titratable acidity determining the quality of fruit were also improved at elevated conditions of EC_700_ compared to ambient as well as open field conditions. Hence under changing climate scenarios tomato production will be benefitted and increase in production may be expected.

## Figures and Tables

**Table 1:** Plant height (cm), number of leaves per plant, canopy spread (cm^2^) and leaf area (cm^2^) of tomato as influenced by elevated CO_2_ and temperature levels.

Sl. No.	Treatments	Plant Height (cm)	Number of Leaves/Plant	Canopy Spread (cm^2^)	Leaf Area (cm^2^)
Mean ± SE					
1	Ambient CO_2_ + Ambient temperature (at OTC)	134.27 ± 6.468 ^a^	127.67 ± 1.950 ^c^	3586.27 ± 54.783 ^bc^	5382.56 ± 82.221 ^d^
2	Elevated CO_2_ (550 ppm)	148.67 ± 2.649 ^a^	147.33 ± 3.066 ^ab^	5748.00 ± 249.040 ^a^	8660.76 ± 180.287 ^a^
3	Elevated CO_2_ (550 ppm) + 2 °C	145.73 ± 1.227 ^a^	144.73 ± 3.013 ^ab^	4079.00 ± 84.911 ^b^	7488.56 ± 155.887 ^b^
4	Elevated CO_2_ (700 ppm)	152.20 ± 3.029 ^a^	158.67 ± 2.423 ^a^	6127.70 ± 75.624 ^a^	9110.68 ± 139.167 ^a^
5	Elevated CO_2_ (700 ppm) + 2 °C	137.87 ± 5.139 ^a^	139.33 ± 3.508 ^bc^	3906.07 ± 98.299 ^b^	6764.23 ± 170.229 ^c^
6	Elevated temperature (+2 °C)	103.47 ± 1.981 ^b^	126.33 ± 3.342 ^c^	3137.00 ± 82.997 ^c^	4149.21 ± 109.778 ^e^
7	Open field	138.13 ± 6.250 ^a^	135.67 ± 3.589 ^bc^	3664.60 ± 96.955 ^bc^	6398.35 ± 169.284 ^c^
	S.Em. ±	3.78	2.96	124.88	145.32
	LSD (*p* = 0.05)	11.64	9.13	384.80	447.77

Note: Values followed by same letter(s) do not differ significantly at *p* = 0.05 level as per Tukeys HSD.

**Table 2:** Number of flowers, fruits per plant, fruit weight (g), yield per plant (kg) and yield per hectare (t) of tomato as influenced by elevated CO_2_ and temperature levels.

Sl. No.	Treatments	Number of Flowers/ Plant	Number of Fruits/Plant	Fruit Weight (g)	Yield per Plant (kg)	Yield per Hectare (t)
(Mean ± SE)						
**1**	Ambient CO_2_ + Ambient temperature (at OTC)	231.35 ± 11.549 ^ab^	228.27 ± 8.315 ^ab^	66.36 ± 1.380 ^d^	3.24 ± 0.088 ^d^	38.17 ± 0.583 ^c^
2	Elevated CO_2_ (550 ppm)	256.73 ± 8.971 ^ab^	256.67 ± 9.929 ^a^	81.34 ± 1.241 ^b^	4.48 ± 0.113 ^b^	52.76 ± 1.100 ^a^
3	Elevated CO_2_ (550 ppm) + 2 °C	254.13 ± 8.288 ^ab^	241.40 ± 5.550 ^ab^	79.40 ± 2.102 ^bc^	4.33 ± 0.089 ^b^	52.09 ± 1.086 ^a^
4	Elevated CO_2_ (700 ppm)	273.80 ± 17.112 ^a^	261.13 ± 10.630 ^a^	90.46 ± 1.381 ^a^	5.09 ± 0.080 ^a^	54.11 ± 0.828 ^a^
5	Elevated CO_2_ (700 ppm) + 2 °C	235.13 ± 4.678 ^ab^	233.87 ± 9.078 ^ab^	73.32 ± 1.847 ^cd^	4.21 ± 0.064 ^b^	46.34 ± 1.164 ^b^
6	Elevated temperature (+2 °C)	223.13 ± 9.936 ^b^	212.80 ± 8.407 ^b^	65.88 ± 1.744 ^d^	2.91 ± 0.062 ^d^	35.65 ± 0.944 ^c^
7	Open field	234.87 ± 5.205 ^ab^	228.40 ± 9.930 ^ab^	68.24 ± 1.422 ^d^	3.67 ± 0.097 ^c^	44.57 ± 1.179 ^b^
	S.Em. ±	9.74	6.97	1.60	0.09	9.81
	LSD (*p* = 0.05)	30.03	21.48	4.92	0.27	30.23

Note: Values followed by same letter(s) do not differ significantly at *p* = 0.05 level as per Tukeys HSD.

**Table 3:** Quality parameters of tomato as influenced by elevated atmospheric CO_2_ and temperature levels.

Sl. No.	Treatment	Keeping Quality (Days)	TSS (°Brix)	Reducing Sugars (%)	Non-Reducing Sugar (%)	Total Sugar (%)
(Mean ± SE)						
1	Ambient CO_2_ + Ambient temperature (at OTC)	15.60 ± 0.412 ^d^	2.83 ± 0.076 ^c^	2.21 ± 0.045 ^b^	1.80 ± 0.036 ^bc^	4.01 ± 0.083 ^ab^
2	Elevated CO_2_ (550 ppm)	24.20 ± 0.370 ^a^	3.13 ± 0.066 ^b^	2.17 ± 0.054 ^b^	2.09 ± 0.054 ^a^	4.26 ± 0.107 ^a^
3	Elevated CO_2_ (550 ppm) + 2 °C	20.60 ± 0.519 ^b^	3.10 ± 0.076 ^bc^	2.47 ± 0.036 ^a^	1.78 ± 0.025 ^bc^	4.25 ± 0.064 ^a^
4	Elevated CO_2_ (700 ppm)	25.60 ± 0.392 ^a^	3.67 ± 0.074 ^a^	2.48 ± 0.036 ^a^	1.93 ± 0.031 ^ab^	4.41 ± 0.068 ^a^
5	Elevated CO_2_ (700 ppm) + 2 °C	18.20 ± 0.380 ^c^	3.07 ± 0.049 ^bc^	2.16 ± 0.045 ^b^	1.97 ± 0.042 ^ab^	4.13 ± 0.086 ^ab^
6	Elevated temperature (+2 °C)	11.60 ± 0.306 ^e^	2.23 ± 0.059 ^d^	2.10 ± 0.055 ^b^	1.64 ± 0.044 ^c^	3.74 ± 0.097 ^b^
7	Open field	17.80 ± 0.369 ^c^	3.03 ± 0.046 ^bc^	2.22 ± 0.059 ^b^	1.87 ± 0.050 ^b^	4.10 ± 0.109 ^ab^
	S.Em. ±	0.41	0.06	0.04	0.05	0.09
	LSD (*p* = 0.05)	1.25	0.20	0.13	0.15	0.27

Note: Values followed by same letter(s) do not differ significantly at *p* = 0.05 level as per Tukeys HSD.

**Table 4:** Quality characteristics of tomato as influenced by elevated atmospheric CO_2_ and temperature levels (cont.,).

Sl. No.	Treatment	Titratable Acidity (%)	Ascorbic Acid (mg/100 g)	pH	Carotenoid co ntent (μg/g)	Lycopene con tent (mg/100 g)
(Mean ± SE)						
1	Ambient CO_2_ + Ambient temperature (at OTC)	0.18 ± 0.006 ^c^	13.10 ± 0.346 ^c^	4.27 ± 0.086 ^ab^	12.12 ± 0.252 ^b^	7.11 ± 0.188 ^cd^
2	Elevated CO_2_ (550 ppm)	0.26 ± 0.003 ^a^	15.80 ± 0.241 ^b^	4.10 ± 0.064 ^ab^	14.53 ± 0.220 ^a^	9.36 ± 0.141 ^a^
3	Elevated CO_2_ (550 ppm) + 2 °C	0.23 ± 0.007 ^b^	15.80 ± 0.241 ^b^	4.17 ± 0.112 ^ab^	13.70 ± 0.364 ^a^	7.96 ± 0.212 ^b^
4	Elevated CO_2_ (700 ppm)	0.27 ± 0.007 ^a^	18.88 ± 0.393 ^a^	4.08 ± 0.104 ^b^	10.70 ± 0.270 ^c^	6.85 ± 0.143 ^de^
5	Elevated CO_2_ (700 ppm) + 2 °C	0.19 ± 0.003 ^c^	15.03 ± 0.312 ^b^	4.18 ± 0.112 ^b^	10.46 ± 0.271 ^c^	6.24 ± 0.154 ^e^
6	Elevated temperature (+2 °C)	0.18 ± 0.003 ^c^	12.33 ± 0.312 ^c^	4.55 ± 0.095 ^a^	9.59 ± 0.146 ^c^	5.15 ± 0.076 ^f^
7	Open field	0.19 ± 0.006 ^c^	13.10 ± 0.346 ^c^	4.25 ± 0.064 ^ab^	12.53 ± 0.332 ^b^	7.58 ± 0.158 ^bc^
	S.Em. ±	0.005	0.31	0.09	0.25	0.15
	LSD (*p* = 0.05)	0.01	0.95	0.28	0.78	0.46

Note: Values followed by same letter(s) do not differ significantly at *p* = 0.05 level as per Tukeys HSD.

**Table 5:** Relationship between yield and quality parameters of tomato under elevated CO_2_, temperature and their combinations.

	Fruit Yield	TSS^#^	TA	RS	NRS	AA	Ph	C
Fruit Yield	1.00							
TSS	0.89 ***	1.00						
TA	0.84 ***	0.73 ***	1.00					
RS	0.64 ***	0.64 ***	0.55 **	1.00				
NRS	0.67 ***	0.63 ***	0.53 **	0.15	1.00			
AA	0.94 ***	0.87 ***	0.88 ***	0.68 ***	0.51 **	1.00		
Ph	−0.67 ***	−0.75 ***	−0.57 ***	−0.35	−0.55 **	−0.65 ***	1.00	
C	0.30	0.28	0.38 *	0.23	0.45 *	0.14	−0.34	1.00

TSS: Total Soluble Solids, TA: Titrable Acidity, RS: Reducing Sugars, NRS: Non-Reducing Sugars, AA: Ascorbic Acid, C: Carotenoids. ***, **, * indicates the significance of Pearson Correlation Coefficient at *p* = 0.001, 0.05, 0.01 respectively as per t-test.

## Data Availability

Not applicable.
